# Pheochromocytoma Diagnosed During First Trimester of Pregnancy

**DOI:** 10.1210/jcemcr/luae027

**Published:** 2024-03-15

**Authors:** Victoria Beard, Maher Ghawji, Fariha Salman, Hooman Oktaei

**Affiliations:** Department of Medicine, The University of Tennessee Health Science Center, Memphis, TN 38163, USA; Department of Medicine, The University of Tennessee Health Science Center, Memphis, TN 38163, USA; Department of Medicine, The University of Tennessee Health Science Center, Memphis, TN 38163, USA; Department of Medicine, The University of Tennessee Health Science Center, Memphis, TN 38163, USA

**Keywords:** pheochromocytoma, pregnancy, hypertensive crisis

## Abstract

Pheochromocytomas are rare catecholamine-secreting tumors that occur in 0.002% of pregnancies. These tumors result in high maternal and fetal morbidity and mortality unless diagnosed in early stages of development, because excess levels of catecholamines cause vasoconstriction of both maternal and uteroplacental vasculature. Paroxysmal hypertension is the most common manifestation, but its variability in presentation and similarity to other pregnancy-related conditions often make diagnosis of pheochromocytoma difficult. Thus, it is essential to consider underlying pathological causes of hypertension during gestation. Diagnosis and treatment of pheochromocytoma must be approached uniquely given the physiologic changes during pregnancy. The standard of care for diagnostic imaging during pregnancy is with magnetic resonance imaging. For these reasons, knowledge of therapy for pheochromocytomas in the pregnant patient is essential for clinical endocrinology practice.

## Introduction

Pheochromocytomas are catecholamine-secreting neuroendocrine tumors that arise from chromaffin cells within the adrenal medulla. The prevalence of pheochromocytomas is approximately 1 in 15 000 to 1 in 54 000 pregnancies per year (1). The majority of pheochromocytomas are sporadic, but there are many associated genetic syndromes, including neurofibromatosis type 1, von Hippel-Lindau syndrome, multiple endocrine neoplasia type 2, and succinate dehydrogenase gene mutations.

Pheochromocytomas may be asymptomatic or may present with episodic headaches, diaphoresis, palpitations, dyspnea, dizziness, nausea, vomiting, anxiety, and abdominal pain and signs including hypertension, tachycardia, pallor, and hyperglycemia. Asymptomatic tumors are incidentally discovered on imaging studies in up to 25% of cases (2). The mainstay of treatment is adequate α-adrenoreceptor blockade followed by adrenalectomy, which has an overall perioperative mortality rate of 3% (2). Even after resection, half of patients will have persistent hypertension and 17% will have recurrent disease, although this is influenced by several factors, including completeness of resection, tumor genetics, and morphology (2). While 10% to 13% of these tumors are considered malignant (3), the determination of malignancy can only be established by the presence of metastasis, as histological features of the tumor are not distinctive.

Diagnosis and treatment of pheochromocytomas during pregnancy can be challenging, since the symptoms may mimic other pregnancy-related conditions, such as preeclampsia, gestational hypertension, or gestational diabetes mellitus. The excess of circulating catecholamines can impact both mother and fetus, increasing the risk of spontaneous abortion, premature labor, intrauterine growth restriction, and fetal hypoxia. Adrenalectomy is the mainstay of treatment in pregnant patients, although gestational age at diagnosis determines the timing of definitive treatment.

## Case Presentation

A 33-year-old African American woman with a history of hypertension, type 2 diabetes mellitus, and sickle cell trait presented to our hospital's emergency department (ED) with complaints of headache and chest pain. She reported a 6-month history of episodic headache, chest pain, palpitations, diaphoresis, tremors, and fatigue. Review of medical records disclosed multiple ED visits for similar complaints over 4 years and multiple instances of hypertensive crisis. Additionally, abdominal imaging from several years prior demonstrated a right suprarenal mass, which had not been investigated further due to frequent patient elopement.

Initial blood pressures (BP) on admission ranged from 178-218/106-132 mmHg with a heart rate (HR) of 107-136 beats per minute (bpm). While the patient's home antihypertensive regimen included amlodipine, losartan, and hydrochlorothiazide, she was intermittently adherent to this regimen. She reported no significant family history and denied use of illicit substances.

## Diagnostic Assessment

Lab investigation demonstrated microcytic anemia, blood glucose 399 mg/dL (22.2 mmol/L) (reference range, 70-170 mg/dL; 3.8-9.5 mmol/L), and troponin 34 ng/L (reference range, ≤ 34 ng/L). Electrocardiography demonstrated sinus tachycardia at a rate of 107 bpm and echocardiography revealed normal cardiac function. Computed tomography (CT) of the chest was ordered to rule out pulmonary embolism but revealed a 3.8-cm soft tissue dense nodularity in the right suprarenal area. Two months later, the patient again presented to the ED with complaints of headache and lower abdominal pain. On this admission, BP ranged 155-164/87-110 mmHg, HR ranged 91-123 bpm. Glucose was 266 mg/dL (14.8 mmol/L), and human chorionic gonadotropin (hCG) was elevated. A transabdominal ultrasound demonstrated intrauterine pregnancy with a gestational age of 11 weeks 5 days.

**Figure 1.**
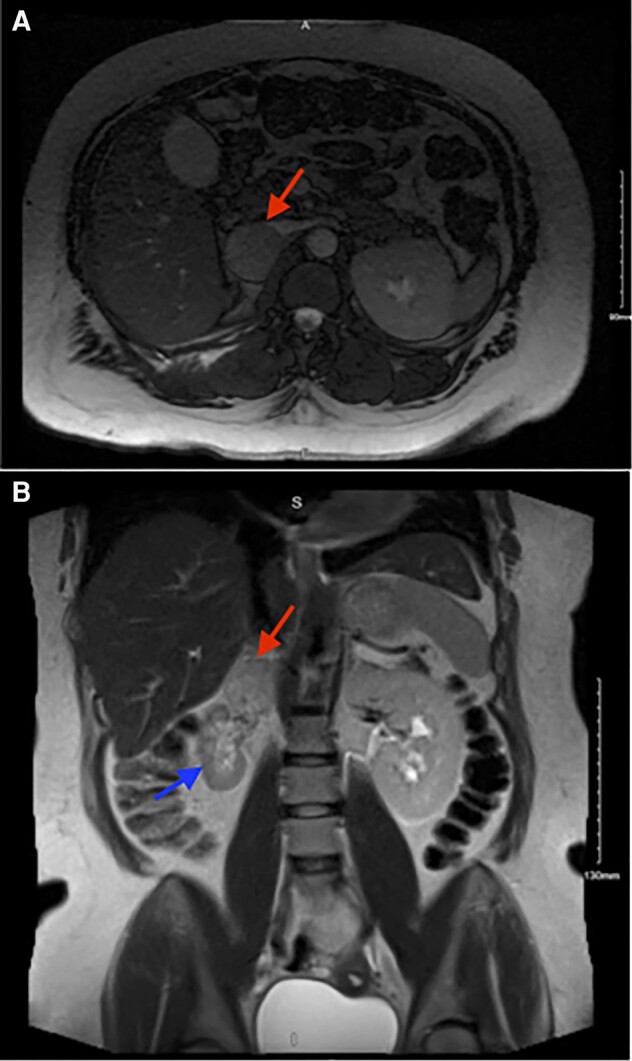
MRI of abdomen and pelvis. (A) Axial section showing 4-cm right adrenal lesion (red arrow) abutting the IVC; (B) Coronal section showing the right adrenal lesion (red arrow) superior to the atrophic right kidney (blue arrow).

Magnetic resonance imaging (MRI) showed a right para-adrenal lesion abutting the inferior vena cava (IVC), most consistent with extra-adrenal neoplasm, mildly increased in size compared to prior CT imaging (Fig. 1). Plasma studies showed normetanephrine levels of 2141.8 pg/mL (12.66 nmol/L) (reference range, 0.00-150.6 pg/mL; 0.00-0.89 nmol/L) and metanephrine 29.58 pg/mL (0.15 nmol/L) (reference range, 0.00-96.64 pg/mL; 0.00-0.49 nmol/L). Levels from 24-hour urine normetanephrine studies were 1739 μg/24 hour (9492 nmol/24 hours) (reference range, 95-650 μg/24 hours; 518.5-3547.9 nmol/24 hour) and norepinephrine levels of 322 μg/24 hours (1903.3 nmol/24 hours) (reference range, 14-120 μg/24 hour; 82.7-709.3 nmol/24 hours) which confirmed the diagnosis of pheochromocytoma.

## Treatment

A multidisciplinary discussion among surgical oncology, obstetrics, cardiology, and endocrinology led to the decision to begin α-blockade with the goal of laparoscopic adrenalectomy prior to 24 weeks gestation. While the patient was placed on phenoxybenzamine 10 mg twice daily, this did not provide adequate BP regulation. The patient was admitted for hypertension control prior to surgery. Over one week, 2 mg doxazosin and 12.5 mg metoprolol twice daily achieved BP control to 117-142/54-81 mmHg and HR 78-101 bpm. Patient underwent laparoscopic right adrenalectomy at 18 weeks 1 day gestation without intraoperative complications. BP was monitored intraoperatively using a noninvasive cuff with ranges 87-232/101-49 mmHg and HR 98-146 bpm. Nicardipine and norepinephrine were administered per anesthesia discretion to regulate labile BP. Fetal heart tones remained within normal range during admission.

## Outcome and Follow-Up

Postoperatively, BP was 123-133/69-80 mmHg and HR 91-103 bpm. Pathology confirmed a 4.2 cm right adrenal pheochromocytoma with moderate differentiation, capsular and lymphovascular invasion, negative tumor margins, and a grading score of adrenal pheochromocytoma and paraganglioma (GAPP) of 5 (range of 0-10). The patient was discharged home with metoprolol 25 mg twice daily for hypertensive control.

Subsequently, the patient presented to the ED 5 days after discharge, with complaints of nausea and hypertension. Her BP ranged from 191-209/99-122 mmHg and HR 114-118 bpm, and she treated with labetalol and ondansetron, but eloped prior to adequate BP control. Two days later, she again presented with headache, nausea, vomiting, and BP of 188-198/116-124 mmHg and HR 113-122 bpm and was treated with labetalol and metoclopramide. Unfortunately, during both visits, she declined transfer to a tertiary care center with a high-risk obstetrician service and has not yet followed up with endocrinology. The child was delivered by emergent C-section at 40 weeks 3 days gestation due to hypertensive crisis at an outside facility.

## Discussion

The excess catecholamines secreted by a pheochromocytoma can significantly impact the health of both mother and fetus. The most common maternal complication is hypertensive crisis, although more serious complications such as arrhythmia, acute coronary syndrome, and acute heart failure may also arise. In rare cases, ischemic stroke, aortic dissection, and pulmonary edema have been reported (4). Overall maternal mortality is 8% to 17% (5, 6). Antepartum diagnosis reduces mortality to less than 1% (5-7), but mortality rises to 29% when diagnosis is delayed to the intra- or postpartum period (6).

Placental enzymes largely prevent catecholamines in the maternal circulation from accessing the fetal blood supply (8). Fetal effects are mediated primarily by vasoconstriction of the uteroplacental circulation, causing insufficient oxygen delivery to the fetus. This increases the risk of spontaneous abortion, premature labor, intrauterine growth restriction, and fetal hypoxia. In cases of pheochromocytoma diagnosed prior to birth, fetal mortality is 9.5% to 15% (5, 6), but delayed detection of pheochromocytoma has a mortality rate of 29% (6).

Presentation of pheochromocytoma during pregnancy may be complex due to its similarity to physiologic changes in pregnancy or other pregnancy-related conditions such as preeclampsia. Thus, one must maintain a high index of suspicion for underlying causes of hypertension, especially during early pregnancy. Normal pregnancy and pheochromocytomas can cause similar symptoms such as nausea or palpitations. Distinguishing features include symptomatology, timing of onset, and laboratory studies. Pheochromocytomas cause paroxysmal hypertension that may present at any point in the pregnancy, while preeclampsia and gestational hypertension often present as consistently elevated BP after 20 weeks gestation. Additionally, pheochromocytomas are not associated with other preeclamptic features such as edema, proteinuria, renal or hepatic dysfunction. In this case, the pheochromocytoma was symptomatic prior to pregnancy with many instances of hypertensive crisis. However, post-conception, this patient's symptoms worsened markedly. While many patients experience worsening effects during the third trimester due to tumor compression by the gravid uterus or uterine contractions, this exacerbation of symptoms in the first trimester is unusual.

Elevated plasma and urine catecholamines are the standard tests for pheochromocytoma; some data suggest that plasma metanephrine levels have the best negative predictive value (4, 8, 9). Importantly, common antihypertensive medications in pregnancy, such as labetalol and methyldopa, can cause falsely elevated catecholamine levels in older lab tests (9). For most patients, the test of choice is CT; whereas in pregnancy, the imaging of choice is MRI without gadolinium to eliminate radiation exposure. Moreover, due to the younger age of the pregnant population, genetic association with syndromic pheochromocytomas must be evaluated, as up to 66% of these tumors presenting during pregnancy may be related to known predisposing mutations (7).

Management of pheochromocytoma is primarily α-adrenergic blockade, then β-blockade and hydration, followed by resection of the tumor. The use of phenoxybenzamine is generally associated with favorable neonatal outcomes, but its ability to cross the placenta can result in neonates becoming hypotensive or experiencing respiratory depression after birth (4). Other common medical strategies include use of doxazosin or prazosin, which may produce fewer adverse effects than phenoxybenzamine due to their shorter half-lives and more selective targeting of the α_1_ receptor (8). Sufficient α-blockage prior to adrenalectomy reduces both maternal and fetal mortality, from 9.5% to 0% and from 55% to 6%, respectively (9). Other agents such as β-adrenergic antagonists, calcium channel blockers, or magnesium sulfate may be added for adequate BP regulation as well as control of tachycardia. In this patient, the low dose of phenoxybenzamine taken as an outpatient did not provide adequate regulation of BP. Given difficulty with medication adherence, the patient was admitted for proper BP management in the hospital. Upon admission and discussion with inpatient pharmacy staff, her inpatient regimen was changed to doxazosin after review of literature showed similar efficacy to phenoxybenzamine, along with increased cost effectiveness and selectivity (1, 10). Doxazosin dosing was not increased due to patient reports of dizziness and systolic BP being well controlled prior to surgery. However, upon reflection, increased fluid and salt intake could have been implemented to allow for increased doxazosin dosage. This may have provided superior α-blockade and improved intraoperative blood pressure control. Metoprolol was added for β-blockade and control of tachycardia per cardiology recommendations.

Gestational age at diagnosis determines the timing of operative resection. If a patient is diagnosed early in gestation, adrenalectomy is preferred prior to 24 weeks gestation to prevent tumor compression by the growing gravid uterus as the pregnancy progresses. However, if the pheochromocytoma is not diagnosed until the third trimester, resection is generally deferred to the postpartum period. This reasoning is due to studies revealing no difference in the mortality rate for antepartum resection vs medical management with postpartum resection (6, 7). Intraoperatively, manipulation of the tumor during excision may trigger release of catecholamine, resulting in labile hypertension. In this case, the patient was diagnosed early in pregnancy, although the adrenal mass predated conception. Her repeated presentation for hypertensive crisis was concerning for imminent threats to her life and that of the fetus. Thus, the consensus among our interprofessional medical team was to proceed with adrenalectomy prior to 24 weeks gestation. During surgical resection, the patient's BP fluctuated considerably despite adequate hydration, necessitating the use of both antihypertensive and vasopressive agents to maintain control per the anesthesiologist.

While this patient had a successful resection, her hypertension did not resolve postoperatively. Her 2 subsequent presentations to the ED for hypertensive crisis are not abnormal, as up to 50% of patients will have continued hypertension after tumor removal (2). However, due to lack of patient follow-up, repeat postoperative catecholamine levels were unable to be obtained. Thus, it remains unclear if her persistent hypertension is related to residual tumor burden, an additional undiagnosed paraganglioma, or other pathology.

## Learning Points

- Pheochromocytomas are rare but potentially lethal tumors during pregnancy. The vasoconstrictive effects of excess catecholamines are damaging to both maternal and uteroplacental vasculature, leading to high mortality rates if not recognized and treated.
- Unusual presentations of hypertension during pregnancy, hypertension prior to 20 weeks gestation, and hypertension that is refractory to medical management should prompt further investigation into underlying pathologies.
- Appropriate treatment of pheochromocytoma diagnosed during early pregnancy includes adequate adrenergic blockage followed by adrenalectomy prior to 24 weeks gestation to prevent compression of these tumors by the enlarging gravid uterus in later stages of pregnancy.

## Contributors

All authors made individual contributions to authorship. V.B., M.G., H.O., and F.S. were involved in diagnosis and management of this patient. V.B. and M.G. were responsible for review of medical records, manuscript creation, and manuscript submission. All authors reviewed and approved the final draft.

## Data Availability

Data sharing is not applicable to this article as no datasets were generated or analyzed during the current study.

## Abbreviations

BP: blood pressure
bpm: beats per minute
CT: computed tomography
ED: emergency department
HR: heart rate
IVC: inferior vena cava
MRI: magnetic resonance imaging
